# Depression increases cancer mortality by 23–83%: a meta-analysis of 65 studies across five major cancer types

**DOI:** 10.1007/s11357-025-01676-9

**Published:** 2025-05-02

**Authors:** Zoltan Ungvari, Mónika Fekete, Annamaria Buda, Andrea Lehoczki, János Tibor Fekete, Péter Varga, Anna Ungvari, Balázs Győrffy

**Affiliations:** 1 Vascular Cognitive Impairment, Neurodegeneration and Healthy Brain Aging Program, Department of Neurosurgery, University of Oklahoma Health Sciences Center, Oklahoma City, OK USA; 2 Stephenson Cancer Center, University of Oklahoma, Oklahoma City, OK USA; 3 Oklahoma Center for Geroscience and Healthy Brain Aging, University of Oklahoma Health Sciences Center, Oklahoma City, OK USA; 4 Department of Health Promotion Sciences, College of Public Health, University of Oklahoma Health Sciences Center, Oklahoma City, OK USA; 5 IDoctoral College/Institute of Preventive Medicine and Public Health, International Training Program in Geroscience Semmelweis University, Budapest, Hungary; 6 Institute of Preventive Medicine and Public Health, Semmelweis University, Semmelweis University, Budapest, Hungary; 7 Jozsef Fodor Center for Prevention and Healthy Aging, Semmelweis University, Budapest, Hungary; 8 Health Sciences Division, Doctoral College, Semmelweis University, Budapest, Hungary; 9 Dept. of Bioinformatics, Semmelweis University, 1094 Budapest, Hungary; 10 Cancer Biomarker Research Group, Institute of Molecular Life Sciences, HUN-REN Research Centre for Natural Sciences, 1117 Budapest, Hungary; 11 Dept. of Biophysics, Medical School, University of Pecs, 7624 Pecs, Hungary

**Keywords:** Psycho-oncology, Psychosocial determinants, Anxiety, Depression, Cancer mortality, Survival, Mortality, Breast cancer, Lung cancer, Prostate cancer, Colorectal cancer, Prognosis, Post-diagnosis, Psychological distress, Oncology, Mental health interventions

## Abstract

Depression is a prevalent but often underrecognized comorbidity among cancer patients. Emerging evidence suggests that psychological distress may adversely impact cancer outcomes, but the magnitude of its effect on survival remains unclear. This meta-analysis evaluates the association between depression diagnosed after cancer diagnosis and cancer-specific and all-cause mortality across major cancer types. A systematic search of PubMed, Web of Science, Google Scholar, and the Cochrane Library was conducted to identify cohort studies examining the impact of depression on cancer mortality. Studies were included if they assessed clinically diagnosed depression or depressive symptoms using validated scales and reported hazard ratios (HRs) for mortality outcomes. A random-effects meta-analysis was performed to pool HR estimates, with heterogeneity assessed via Cochran’s *Q* and *I*^2^ statistics. Funnel plots and Egger’s test were used to evaluate publication bias. A total of 65 cohort studies were included. Depression was associated with significantly increased cancer-specific mortality in colorectal cancer (HR 1.83, 95% CI 1.47–2.28), breast cancer (HR 1.23, 95% CI 1.13–1.34), lung cancer (HR 1.59, 95% CI 1.36–1.86), and prostate cancer (HR 1.74, 95% CI 1.36–2.23). When considering mixed cancer types, depression was linked to a 38% increased risk of cancer mortality (HR 1.38, 95% CI 1.20–1.60). Significant heterogeneity was observed across studies (*I*^2^ range 56–98%), suggesting variations in study populations and methodologies. Sensitivity analyses confirmed the robustness of the findings, and trial sequential analysis indicated sufficient evidence for a conclusive association. Depression after cancer diagnosis is associated with a significantly increased risk of cancer-specific mortality across multiple cancer types. These findings highlight the urgent need for integrating routine mental health screening and interventions into oncology care. Future research should focus on mechanistic pathways and targeted interventions to mitigate the negative impact of depression on cancer survival.

## Introduction

Cancer remains a leading cause of morbidity and mortality worldwide, accounting for nearly 10 million deaths annually [1–3]. Advances in early detection, targeted therapies, and supportive care have significantly improved survival outcomes across multiple cancer types. However, beyond biological and clinical factors, increasing attention has been directed toward psychosocial determinants of cancer prognosis [4, 5]. Depression, a prevalent comorbidity among cancer patients, has emerged as a potential predictor of worse survival outcomes [6–9]. The interdisciplinary field of psycho-oncology has emerged to explore these complex interactions, aiming to integrate psychological and social aspects into comprehensive cancer care.

Depression affects up to 20–30% of individuals diagnosed with cancer, manifesting through persistent sadness, fatigue, loss of motivation, and reduced treatment adherence [10–13]. The psychological burden of a cancer diagnosis, combined with the physiological stress of the disease and its treatment, contributes to an increased risk of developing depression [14]. Despite the well-documented effects of depression on quality of life, its impact on cancer progression and mortality remains underappreciated. The clinical relevance of depression following a cancer diagnosis is well established [15]. The diagnosis itself represents a major psychological stressor that can trigger a cascade of emotional responses, ranging from acute distress to diagnosable mood disorders. Studies have shown that a significant proportion of newly diagnosed cancer patients experience adjustment disorders or major depressive episodes within the first year of diagnosis, often in response to fear of death, loss of function, or anticipated suffering [16–21]. Psychological models such as the distress trajectory framework suggest that, in a subset of patients, transient distress may evolve into persistent depression that adversely affects coping, decision-making, and treatment engagement [21]. These psychological burdens can emerge even in early-stage disease and are not limited to those with advanced or terminal cancer. Therefore, identifying and addressing mood disturbances early in the cancer care continuum is of critical importance for improving both quality of life and treatment outcomes [15].

A growing body of evidence suggests that depression may influence cancer survival through multiple biological and behavioral mechanisms [15, 22–25]. Biologically, depression is linked to chronic inflammation, dysregulation of the hypothalamic–pituitary–adrenal (HPA) axis, immune suppression, and alterations in autonomic nervous system function—factors that may accelerate tumor progression [26]. Behaviorally, depressed patients are more likely to experience delays in seeking treatment, exhibit poor adherence to oncologic therapies, and engage in health-risk behaviors, including smoking and physical inactivity, all of which can contribute to poorer survival outcomes.

Several cohort studies and meta-analyses have explored the relationship between depression and cancer mortality, yet inconsistencies remain due to variations in study design, depression assessment methods, and cancer types investigated. While some studies report a clear association between depression and increased cancer mortality, others suggest that the relationship is confounded by socioeconomic status, treatment disparities, or pre-existing health conditions. Additionally, the extent to which depression affects survival across different cancer types remains an area of active research.

To address these gaps, we conducted a comprehensive meta-analysis to evaluate the impact of depression diagnosed after cancer diagnosis on cancer-specific and all-cause mortality. Our study aims to quantify the association between depression and survival in patients with five major cancer types—colorectal, breast, lung, prostate, and mixed cancers—while addressing potential sources of heterogeneity. We hypothesize that depression significantly increases mortality risk across all cancer types and that this effect is independent of traditional prognostic factors. By synthesizing evidence from multiple studies, this analysis provides critical insights into the prognostic role of depression in oncology and underscores the need for integrated psychosocial interventions to improve cancer outcomes.

## Methods

### Data sources and search strategy

**Fig. 1:**
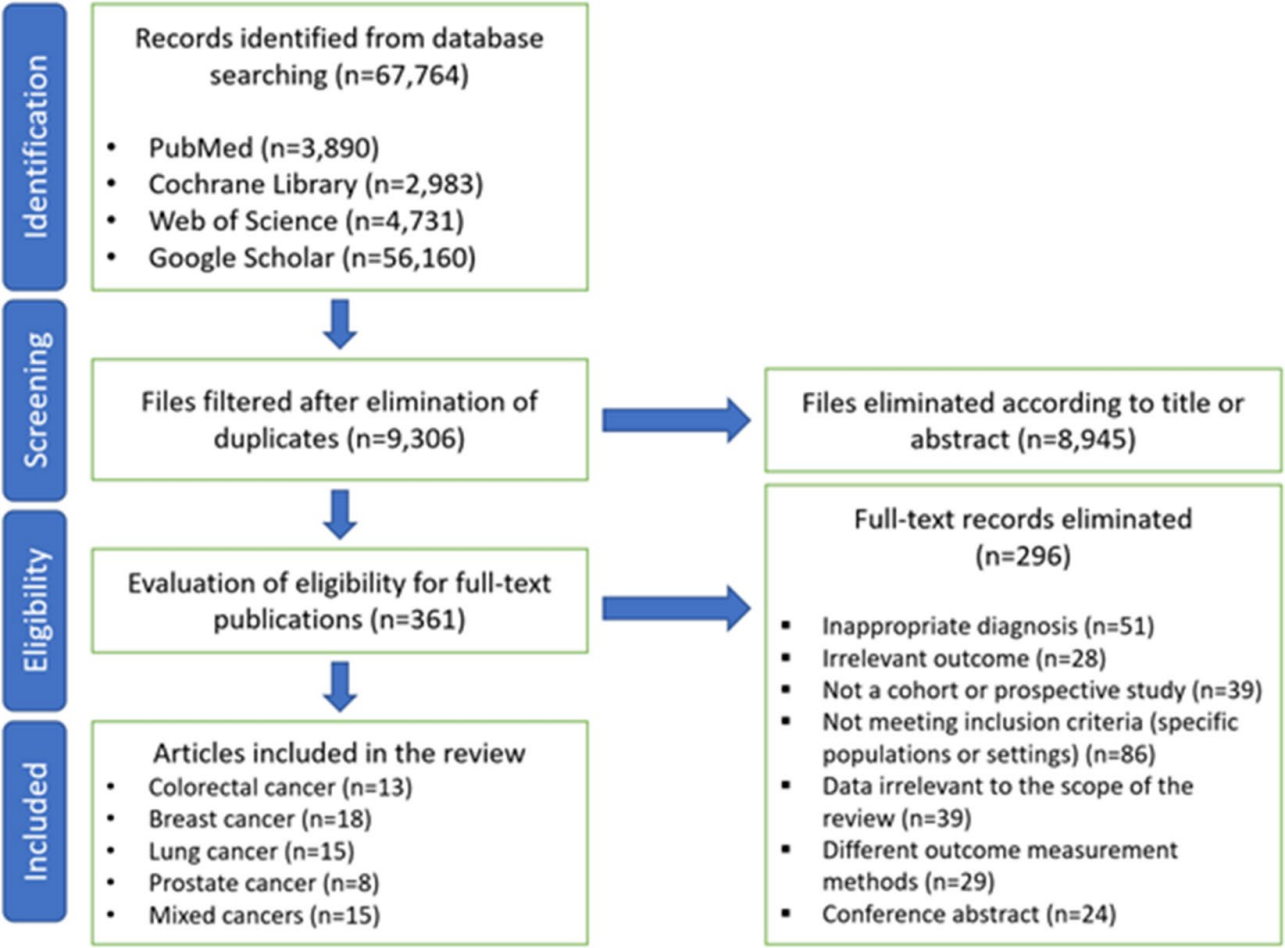
Flowchart of the study selection process. A total of 67,764 records were identified from four databases: PubMed, Cochrane Library, Web of Science, and Google Scholar. After removing duplicates (*n* = 9,306), 361 articles were assessed for full-text eligibility. Following exclusion based on title, abstract, and eligibility criteria, 65 articles were included in the meta-analysis. These articles covered various cancer types, including colorectal cancer, breast cancer, lung cancer, prostate cancer, and mixed cancers

This comprehensive meta-analysis involved a thorough search of multiple online databases, including PubMed, Web of Science, Google Scholar, and the Cochrane Library, covering studies from their inception until October 1, 2024 (Fig. 1). The objective was to identify studies examining the association between depression and the risk of cancer-specific mortality as well as all-cause mortality in cancer patients. The search was restricted to studies published in English and conducted on human subjects. Furthermore, the reference lists of relevant identified publications were manually reviewed to identify additional studies that met the eligibility criteria. No ethical concerns were raised, as all data analyzed were derived from previously published studies.

The search terms used consisted exclusively of Medical Subject Headings (MeSH) terms (Table 1). These terms were selected to focus on the relationship between mental distress, specifically depression, and cancer, with a particular emphasis on mortality outcomes. The MeSH terms targeted mental health conditions (e.g., “Depressive Disorder” OR “Depression”), cancer-related terms (e.g., “Neoplasms” OR “Carcinoma” OR “Malignancy” OR “Tumor”), and outcome measures (e.g., “Mortality” OR “Death”) in studies of cohort or prospective design.

**Table 1:** Search strategy using medical subject headings (MeSH) terms

Search category	MeSH terms
Colon cancer, depression, and mortality	“Colorectal tumor” OR “Colon cancer” AND “Depression” AND “Survival” OR “Mortality”
Breast cancer, depression, and mortality	“Breast Tumor” AND “Depression” AND “Survival Rate” OR “Mortality”
Lung cancer, depression, and mortality	“Depression” AND “Lung Tumor” AND “Mortality” OR “Survival Rate”
Prostate cancer, depression, and mortality	“Prostatic Neoplasms” OR „Prostate Cancer” AND “Depression” AND “Mortality” OR “Survival Rate”

Two reviewers (AL and MF) independently assessed the eligibility of the articles. Any disagreements that were not resolved through discussion between the two reviewers were reviewed by a third author (AU). After removing duplicates, the titles and abstracts of all potentially relevant studies were screened, and clearly unrelated articles were excluded. The full text of the remaining articles was then reviewed, and they were included in the final list if they met the eligibility criteria.

### Eligibility criteria

In our study, we included original cohort studies, both prospective and retrospective, that met the following criteria. We focused on cancer patients enrolled in cohorts to evaluate cancer-specific or all-cause mortality risk.

The studies we selected assessed clinical depression using established diagnostic criteria, such as those outlined in the *Diagnostic and Statistical Manual of Mental Disorders* (DSM) or the International Classification of Diseases (ICD). Additionally, we considered studies that measured psychological distress, specifically symptoms of depression, using validated symptom scales.

To ensure the robustness of our analysis, we only included studies that reported risk estimates for mortality rates. These estimates encompassed measures of association, such as relative risk (RR), hazard ratio (HR), or odds ratio (OR), along with their corresponding measures of uncertainty, typically 95% confidence intervals (CIs).

Given the potential for reverse causality, where diagnosed or undiagnosed cancer might impact mood, we implemented a minimum follow-up duration criterion. All included studies had an average or median follow-up period of at least 2 years. This approach helped mitigate the risk of overestimating the association between depression and cancer outcomes.

### Statistical analysis

Statistical analyses were performed using the MetaAnalysisOnline.com web-based tool [27]. A random-effects model was employed to calculate pooled hazard ratios (HRs) and 95% confidence intervals (CIs), accommodating potential heterogeneity across studies to enhance the generalizability of the results. Forest plots were generated to visualize individual study outcomes alongside the overall pooled effect, providing a clear graphical summary of effect estimates and allowing for the comparison of study-level data and potential result variations.

Heterogeneity was assessed using Cochran’s *Q* (chi-square test) and the *I*^2^ statistic. While Cochran’s *Q* evaluated whether the observed variability in effect sizes exceeded that expected by chance, the *I*^2^ index quantified the proportion of total variability attributable to between-study heterogeneity, rather than sampling error.

### Assessment of publication bias

Potential publication bias was evaluated through funnel plots, which examined the relationship between effect sizes and standard errors to detect asymmetry. Egger’s test was performed to further evaluate bias by analyzing the correlation between effect estimates and their precision.

### Trial sequential analysis

Trial sequential analysis (TSA) was conducted using the *metacoumbounds* package in Stata version 14.1 to evaluate the robustness of the cumulative sample size and to determine if the evidence was sufficient for reliable conclusions. A relative risk reduction of 15%, two-sided *α* of 5%, and 80% power were assumed to calculate the required a priori information size (APIS), representing the minimum participant number needed to detect a statistically significant effect.

### Cancer-type specific analysis

Statistical analyses were carried out separately within five independent disease cohorts representing the largest groups of cancer patients: those with lung cancer, breast cancer, colorectal cancer, prostate cancer, and, in the final setting, mixed cancers.

## Results

### Colorectal cancer mortality

**Fig. 2:**
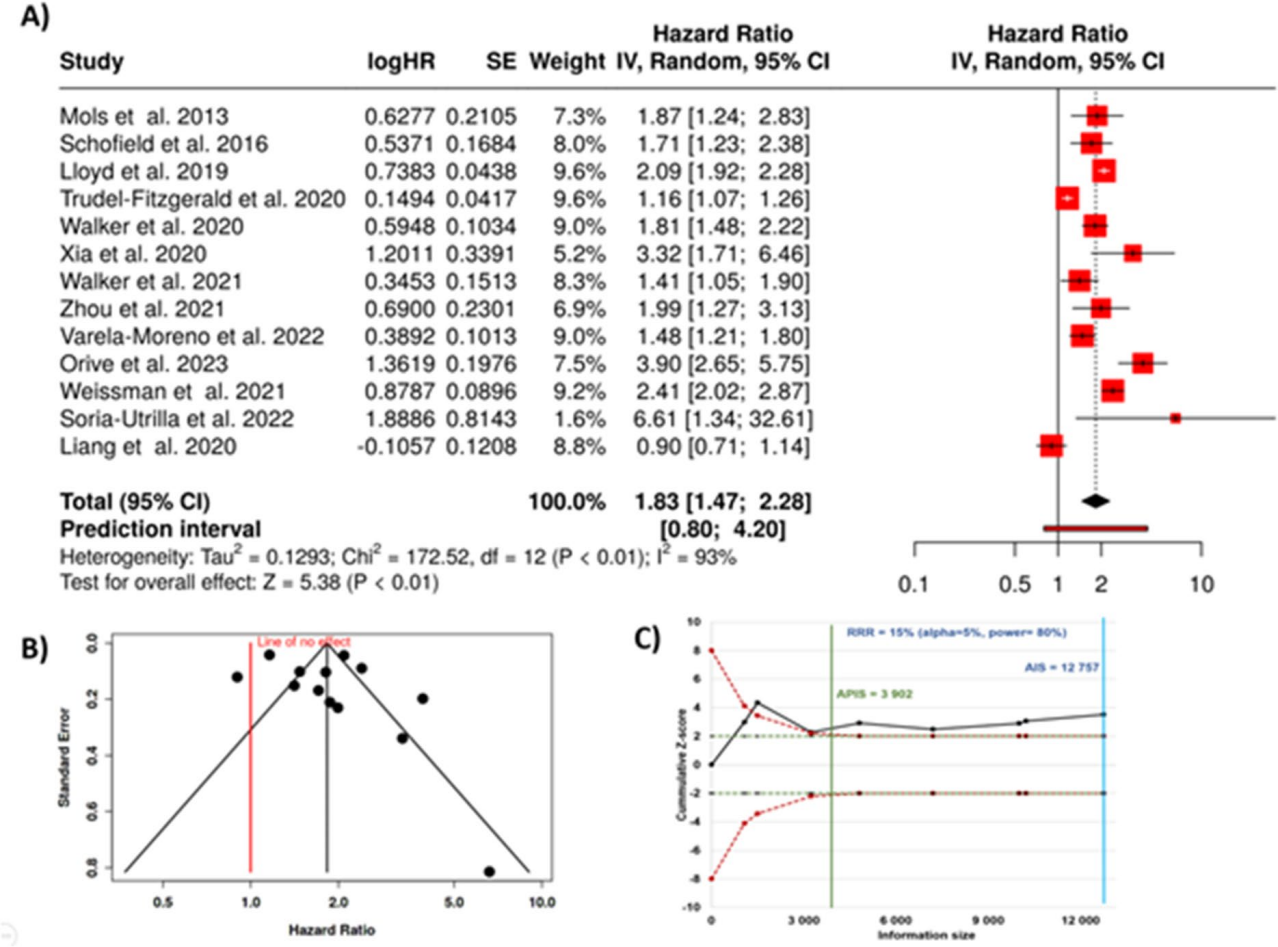
Meta-analysis results for the association between depression and colorectal cancer mortality. **A** Forest plot displaying hazard ratios with 95% confidence intervals for individual studies and the pooled random-effects estimate (HR 1.83). Significant heterogeneity was observed across studies (*I*^2^ = 93%, *p* < 0.01). **B** Funnel plot assessing potential publication bias. C Trial sequential analysis showing the cumulative Z-curve (black) crossing the trial sequential monitoring boundary for benefit, indicating sufficient evidence for an association between depression and CRC mortality. HR, hazard rate; SE, standard error; CI, confidence interval; IV, inverse variance; AIS, actual information size; APIC, a priori information size; RRR, relative risk reduction

In total, 13 cohorts were included in this meta-analysis [28–40]. Using the random-effects model and the inverse variance method to compare the hazard rate, we observed a statistically significant association between depression and colorectal cancer mortality. The summarized HR was 1.83, with a 95% confidence interval of 1.47 to 2.28, indicating an 83% higher risk of cancer mortality among those with depression compared to those without (as presented in Fig. 2A).

The overall effect assessment showed statistical significance at a *p*-value of less than 0.05. However, there was significant heterogeneity across the included studies (*p* < 0.01), suggesting that the variation in effect sizes may be due to differences in the characteristics of the cohorts rather than random chance. The *I*^2^ statistic revealed that 93% of the inconsistency was attributable to true heterogeneity, not sampling variability.

Despite this heterogeneity, the funnel plot did not reveal evidence of publication bias. Furthermore, Egger’s test for funnel plot asymmetry was not significant, with an intercept of 1.68 (95% CI − 1.77 to 5.13), *t*-value of 0.956, and *p*-value of 0.36, indicating no significant small-study effects (Fig. 2B). The trial sequential analysis validated that the accumulated data surpassed the required threshold for drawing definitive conclusions (Fig. 2C).

### Breast cancer mortality

**Fig. 3:**
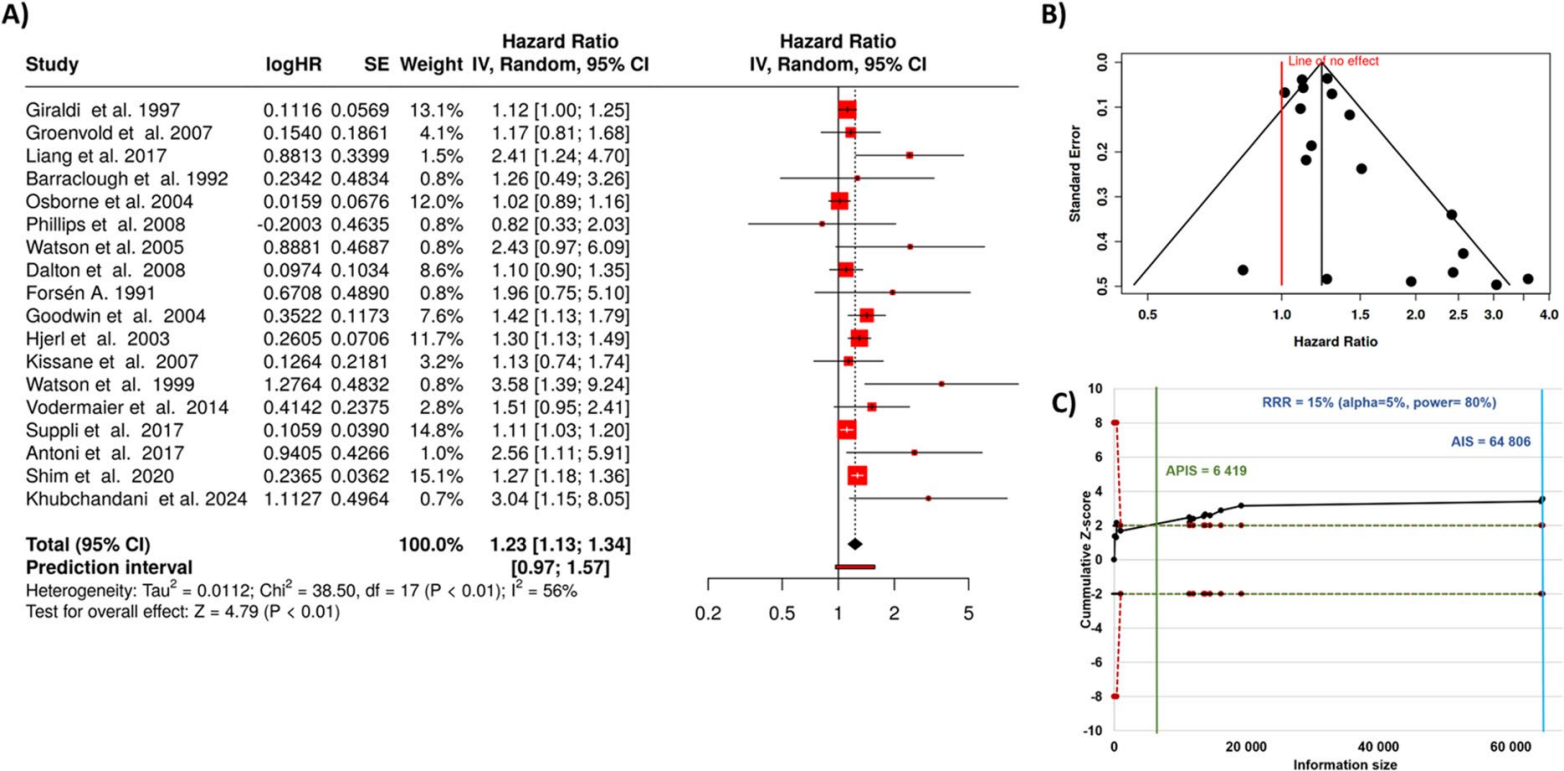
Association between depression and breast cancer mortality. **A** Forest plot showing study-specific and pooled hazard ratios with 95% confidence intervals. Random-effects meta-analysis yielded a pooled HR of 1.23, with significant between-study heterogeneity (*I*^2^ = 56%, *p* < 0.01). **B** Funnel plot for assessment of publication bias. **C** Trial sequential analysis demonstrating that the cumulative Z-curve (black line) crosses the monitoring boundary, indicating robust evidence for increased mortality risk among depressed breast cancer patients. HR, hazard rate; SE, standard error; CI, confidence interval; IV, inverse variance; AIS, actual information size; APIC, a priori information size; RRR, relative risk reduction

When evaluating the correlation between depression and mortality in breast cancer, a total of 18 studies were included [41–58]. Using a random-effects model and the inverse variance method, we found a statistically significant association between depression and cancer mortality. The pooled analysis revealed a hazard rate of 1.23, with a 95% confidence interval ranging from 1.13 to 1.34, suggesting a 23% increased risk of cancer mortality in individuals with depression compared to those without (as depicted in Fig. 3A).

The overall effect was statistically significant, with a *p*-value of less than 0.05. However, there was considerable heterogeneity among the studies (*p* < 0.01), indicating variability in the size or direction of the observed effects. This heterogeneity was quantified by an *I*^2^ value, which showed that 56% of the variability across studies was due to true differences in study results, rather than random variation.

Further assessment of the funnel plot indicated the possibility of publication bias, which was confirmed by Egger’s test. Egger’s analysis demonstrated significant funnel plot asymmetry, with an intercept of 1.07, a confidence interval of 0.18 to 1.95, a *t*-value of 2.354, and a *p*-value of 0.032, suggesting that smaller studies may have disproportionately influenced the overall effect estimate (Fig. 3B). Sequential trial analysis demonstrated adequate sampling power, supporting the reliability of our findings (Fig. 3C).

### Lung cancer mortality

**Fig. 4:**
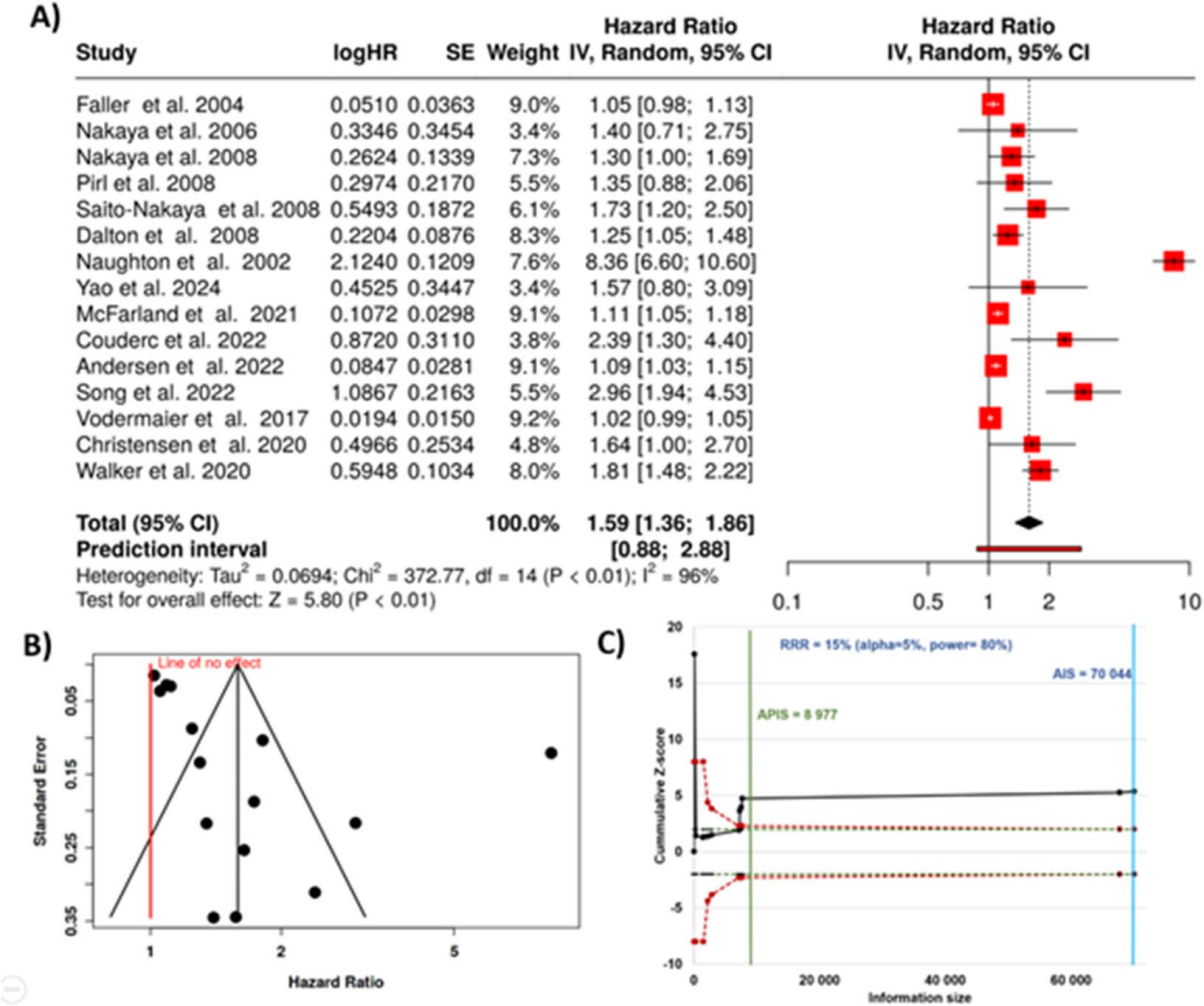
Correlation between depression and lung cancer mortality. **A** Forest plot displaying study-specific and pooled hazard ratios with 95% confidence intervals. Random-effects meta-analysis yielded a combined HR of 1.59. **B** Funnel plot for assessment of publication bias. C Trial sequential analysis demonstrating that the cumulative Z-curve (black line) crosses the monitoring boundary, indicating robust evidence for increased mortality risk among depressed lung cancer patients. HR, hazard rate; SE, standard error; CI, confidence interval; IV, inverse variance; AIS, actual information size; APIC, a priori information size; RRR, relative risk reduction

In this meta-analysis, a total of 15 studies were included to evaluate the association between depression and lung cancer mortality [33, 48, 59–71]. Using a random-effects model with the inverse variance method, the analysis demonstrated a statistically significant association. The pooled hazard rate indicated that individuals with depression had a 59% higher risk of lung cancer mortality compared to those without depression, with a summary estimate of 1.59 and a 95% confidence interval ranging from 1.36 to 1.86 (visually represented in Fig. 4A).

The test for the overall effect was statistically significant, with a *p*-value of less than 0.05, confirming the robustness of this association. However, significant heterogeneity was observed across the studies (*p* < 0.01), suggesting considerable variability in the magnitude or direction of the effect estimates. This heterogeneity was quantified by an *I*^2^ value of 96%, indicating that nearly all of the variability between studies could be attributed to true differences in effect sizes rather than random variation.

Further examination of the funnel plot suggested the possibility of publication bias, which was supported by Egger’s test for funnel plot asymmetry (Fig. 4B). Egger’s test showed a significant result, with an intercept of 4.05, a confidence interval between 1.22 and 6.88, a *t*-value of 2.801, and a *p*-value of 0.015. This finding indicates that smaller studies with more pronounced effects may have skewed the overall estimate, underscoring the need for caution when interpreting these results.

Finally, the TSA analysis confirmed that the cumulative evidence exceeded the threshold needed for robust statistical inference (Fig. 4C).

### Prostate cancer mortality

**Fig. 5:**
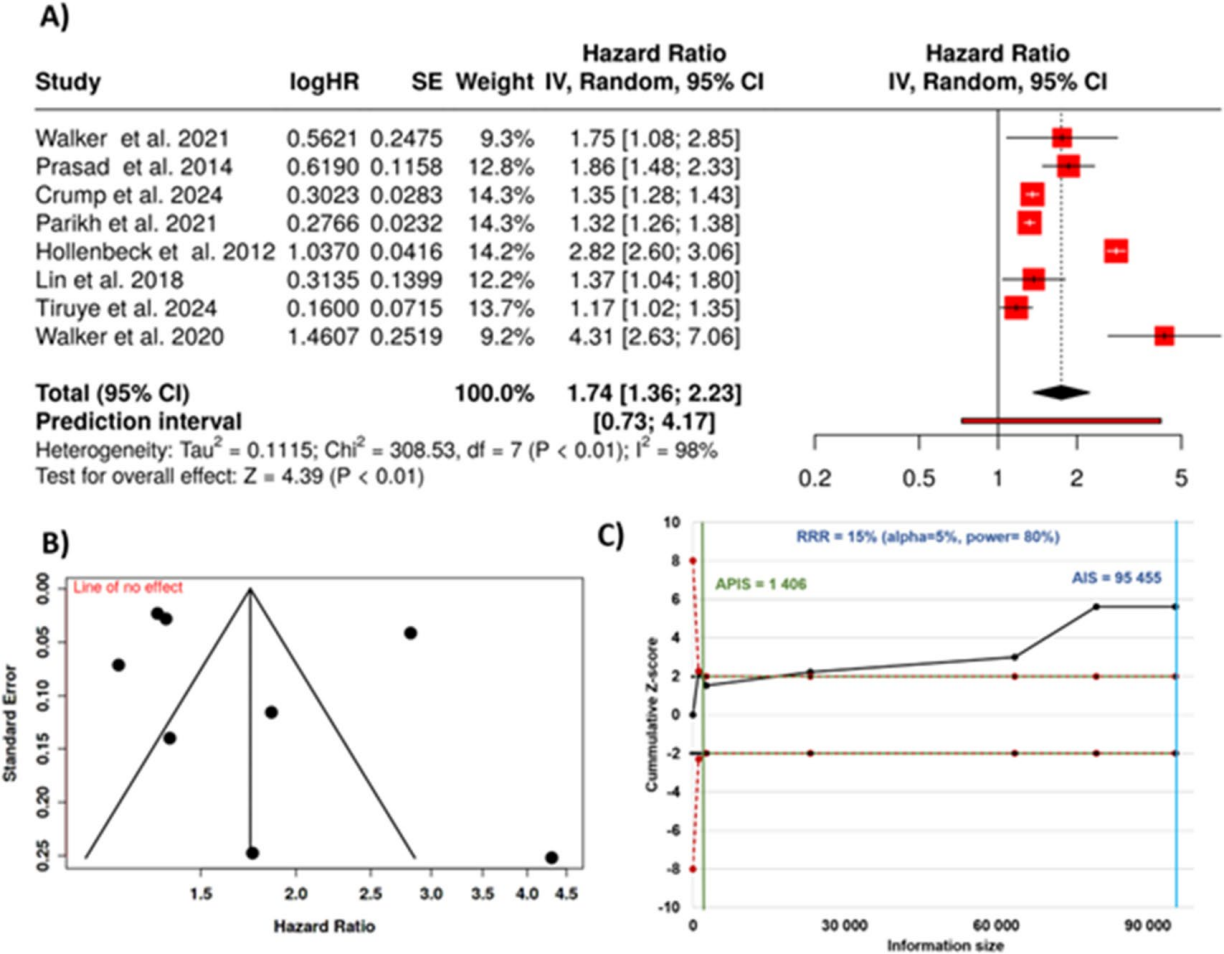
Relationship between psychological depression and death rates in prostate cancer patients. **A** Forest diagram illustrating individual and aggregated risk estimates (hazard ratios) with corresponding uncertainty ranges (95% confidence intervals). Pooled analysis using random-effects modeling revealed a combined risk estimate of 1.74. **B** Publication bias assessment via symmetry visualization plot. **C** Sequential trial evaluation showing the accumulated Z-curve path (black line) surpassing the monitoring threshold, suggesting substantial evidence for elevated mortality among prostate cancer patients with depressive symptoms. HR, hazard rate; SE, standard error; CI, confidence interval; IV, inverse variance; AIS, actual information size; APIC, a priori information size; RRR, relative risk reduction

In this analysis, eight studies were included to examine the relationship between depression and prostate cancer mortality [33, 35, 72–77]. The analysis, conducted using a random-effects model with the inverse variance method, revealed a statistically significant association between depression and increased mortality in prostate cancer patients. The pooled hazard rate indicated that individuals with depression had a 74% higher risk of dying from prostate cancer compared to those without depression, with a summary estimate of 1.74 and a 95% confidence interval ranging from 1.36 to 2.23. These findings suggest that depression may significantly increase the risk of prostate cancer mortality (Fig. 5A).

The overall effect was statistically significant, with a *p*-value of less than 0.05, supporting the robustness of this association. However, substantial heterogeneity was detected among the studies (*p* < 0.01), implying that the magnitude and/or direction of the effect varied considerably across the studies. The *I*^2^ statistic confirmed that 98% of the observed variability was due to genuine differences in study outcomes, rather than random chance, highlighting the presence of significant heterogeneity.

Despite this heterogeneity, no evidence of publication bias was observed. The funnel plot did not suggest any asymmetry, and Egger’s test further supported this conclusion. Egger’s test yielded an intercept of 2.97, with a confidence interval of − 4.62 to 10.55, a *t*-value of 0.766, and a *p*-value of 0.473, indicating that small-study effects were unlikely to have influenced the results (Fig. 5B). Overall, the absence of publication bias strengthens confidence in the overall association between depression and prostate cancer mortality. Figure 5C shows that the trial sequential analysis confirmed that the aggregated sample size provided sufficient statistical power to support conclusive findings.

### Mixed tumors and mortality

**Fig. 6:**
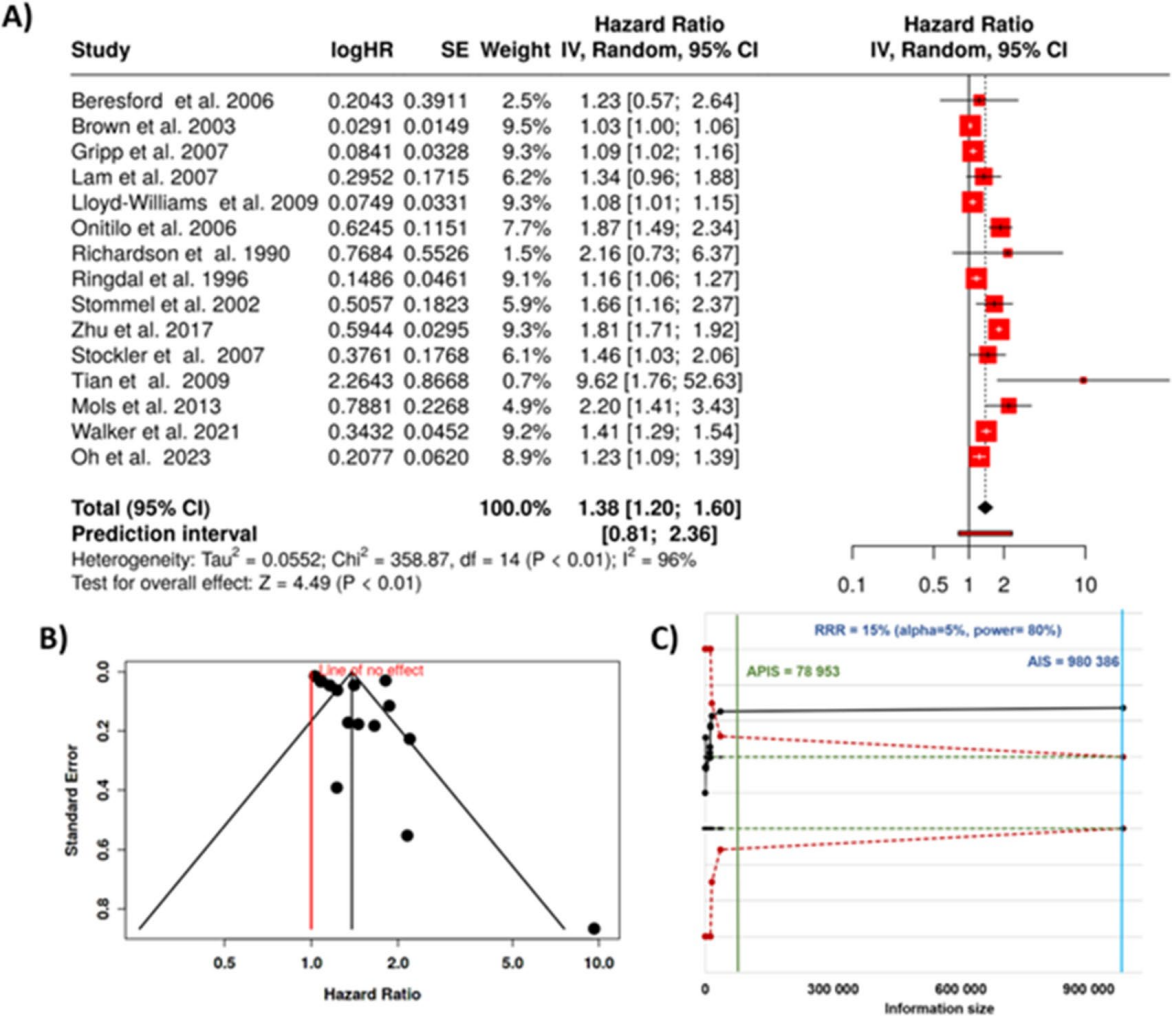
Association between depression and cancer mortality in mixed tumor types. **A** Forest plot showing study-specific and pooled hazard ratios with 95% confidence intervals. Random-effects meta-analysis yielded a pooled HR of 1.38, with significant between-study heterogeneity (*I*^2^ = 96%, *p* < 0.01). **B** Funnel plot for the assessment of publication bias. **C** Trial sequential analysis demonstrating that the cumulative Z-curve (black line) crosses the monitoring boundary, indicating robust evidence for increased mortality risk among depressed cancer patients. HR, hazard rate; SE, standard error; CI, confidence interval; IV, inverse variance; AIS, actual information size; APIC, a priori information size; RRR, relative risk reduction

In this final analysis, 15 cohort studies were analyzed to assess the relationship between depression and mortality across a combined cohort of various cancer types [28, 35, 78–90]. The analysis, performed using a random-effects model with the inverse variance method, revealed a statistically significant association between depression and increased cancer mortality. The pooled hazard rate indicated that individuals with depression had a 38% higher risk of mortality from cancer compared to those without depression. The summarized hazard rate was 1.38, with a 95% confidence interval ranging from 1.20 to 1.6 (Fig. 6A).

The overall effect was statistically significant, with a *p*-value below 0.05, reinforcing the association between depression and elevated cancer mortality. However, significant heterogeneity was detected among the included cohorts (*p* < 0.01), suggesting considerable variation in the magnitude or direction of the effect across different studies. The *I*^2^ statistic showed that 96% of this variability was due to true heterogeneity rather than random chance, indicating substantial differences in study outcomes that could be related to the cancer type, population characteristics, or study methodologies.

Despite this heterogeneity, the funnel plot did not reveal evidence of publication bias. Egger’s test for funnel plot asymmetry also confirmed the absence of small-study effects, with an intercept of 2.9, a confidence interval between − 0.49 and 6.29, a *t*-value of 1.677, and a *p*-value of 0.117, suggesting that the results were not unduly influenced by the selective reporting of studies (Fig. 6B). Using trial sequential methodology, we verified that the total number of analyzed cases reached a level that substantiates the strength and dependability of our conclusions (Fig. 6C).

These findings suggest that depression is associated with a significantly higher risk of mortality across various cancer types. The observed heterogeneity implies that the effect of depression on cancer outcomes may vary depending on the specific cancer type, patient populations, or other factors. However, the absence of publication bias supports the validity of the overall association. Further research is needed to explore the underlying mechanisms driving this relationship and to understand the role of depression in specific cancer subtypes.

## Discussion

This meta-analysis provides compelling evidence that depression diagnosed after a cancer diagnosis is associated with significantly increased cancer-specific and all-cause mortality across multiple cancer types. Our findings demonstrate that depression is linked to an 83% increased risk of mortality in colorectal cancer, a 59% increased risk in lung cancer, a 74% increased risk in prostate cancer, and a 23% increased risk in breast cancer. Furthermore, the overall pooled analysis across mixed cancer types suggests a 38% higher risk of mortality among patients with depression. These results highlight the crucial role of mental health in cancer prognosis and emphasize the need for integrating psychological support into routine oncologic care.

Several biological and behavioral pathways may underlie the observed association between depression and increased cancer mortality [22, 23]. Biologically, depression has been linked to chronic systemic inflammation [91], dysregulation of the hypothalamic–pituitary–adrenal (HPA) axis, immune suppression, and autonomic dysfunction—all of which may contribute to tumor progression [26]. Elevated levels of pro-inflammatory cytokines, including interleukin-6 (IL-6) and tumor necrosis factor-alpha (TNF-α), have been reported in depressed individuals and are known to play a role in cancer progression and metastasis [91, 92]. Notably, some of the biological mechanisms implicated in the link between depression and cancer progression—such as chronic inflammation and immune suppression—may appear contradictory at first glance. However, these phenomena can coexist and are increasingly recognized as components of a dysregulated immune phenotype commonly observed in aging. This paradox of simultaneous immune activation and functional suppression has been termed “inflammaging” [93] and is considered a hallmark of biological aging and age-related disease vulnerability, including cancer [94]. Thus, depression may exacerbate age-associated immune dysfunction, creating a tumor-permissive environment through both inflammatory signaling and impaired immune surveillance. Additionally, depression is associated with increased oxidative stress and impaired DNA repair mechanisms, potentially accelerating oncogenesis and reducing treatment efficacy [95–97].

From a behavioral perspective, depression may impact cancer outcomes by influencing health behaviors and treatment adherence [98]. Depressed patients are more likely to experience delays in seeking medical care, have lower adherence to chemotherapy, radiotherapy, and hormonal therapies, and engage in unhealthy behaviors such as smoking, poor nutrition, and physical inactivity [99]. These factors collectively contribute to worse treatment responses and overall prognoses. Furthermore, depressed individuals often face social isolation and reduced support systems, which may further exacerbate their vulnerability to poor health outcomes [99, 100].

Despite the strong overall association between depression and cancer mortality, significant heterogeneity was observed across studies. This variation may be attributed to differences in study populations, methods of depression assessment (e.g., clinical diagnosis vs. self-reported symptom scales), cancer stages, treatment types, and follow-up durations. For instance, the impact of depression on survival may be more pronounced in cancers with longer disease courses, such as breast and prostate cancer, where sustained psychological distress may have a cumulative effect on health behaviors and treatment adherence.

The findings of this study underscore the need for routine depression screening and early intervention in oncology settings. Given the strong association between depression and mortality, integrating psychosocial care into standard oncologic treatment could improve patient outcomes. Psychological interventions, such as cognitive-behavioral therapy (CBT) [101–103], supportive-expressive therapy [104, 105], and mindfulness-based interventions [9], have shown promise in reducing distress and improving adherence to cancer treatment. Moreover, pharmacological management of depression, including the use of selective serotonin reuptake inhibitors (SSRIs) and other antidepressants, may play a role in improving mental health and potentially influencing cancer progression, though further research is needed in this area [106–109]. Notably, in certain cancers, patients using SSRIs exhibited a significantly shorter time to disease progression [110]. This may be attributed to the potential of SSRIs to alter serotonin levels within the tumor microenvironment, thereby activating proliferation pathways.

While our meta-analysis provides robust evidence supporting the link between depression and cancer mortality, several limitations must be acknowledged. First, the observational nature of the included studies precludes causal inference, and residual confounding by unmeasured variables (e.g., socioeconomic status, access to care, genetic predisposition) cannot be entirely ruled out. Second, the heterogeneity across studies, while accounted for using a random-effects model, suggests that differences in study designs and patient populations may have influenced the findings. Third, the methods of depression assessment varied, with some studies relying on self-reported questionnaires, while others used clinical diagnostic criteria, potentially introducing measurement bias. Future research should focus on longitudinal studies that assess the temporal relationship between depression and cancer progression while accounting for potential confounders. Additionally, randomized controlled trials (RCTs) investigating the impact of targeted depression interventions on cancer survival outcomes are needed. Identifying the most effective psychological and pharmacological treatments [107] for depression in cancer patients could inform clinical guidelines and improve multidisciplinary cancer care.

The biological pathways linking depression to cancer mortality—chronic inflammation, immune suppression, and dysregulation of stress response systems—are also key drivers of aging [111–113]. Cancer itself is an age-related disease, and many of its underlying pathogenetic mechanisms overlap with those of biological aging [113]. It is plausible that depression further accelerates these aging processes, compounding the risk of cancer progression [114]. Future research should explore how depression-induced alterations in inflammatory, metabolic, and cellular stress pathways interact with aging biology to influence cancer outcomes.

This meta-analysis confirms that depression significantly increases cancer mortality across multiple cancer types, underscoring the critical role of mental health in cancer prognosis. These findings highlight the urgent need for integrating mental health screening and interventions into oncology care to improve survival outcomes. Addressing depression in cancer patients should be considered a priority for oncologists, mental health professionals, and healthcare policymakers to optimize cancer treatment and enhance overall well-being.
